# Control of the rhizobium–legume symbiosis by the plant nitrogen demand is tightly integrated at the whole plant level and requires inter-organ systemic signaling

**DOI:** 10.3389/fpls.2023.1114840

**Published:** 2023-03-09

**Authors:** Marc Lepetit, Renaud Brouquisse

**Affiliations:** Institut Sophia Agrobiotech, Institut National de Recherche pour l’Agriculture (INRAE), l’alimentation et l’Environnement, Université Côte d’Azur, Centre National de Recherche Scientifique (CNRS), Sophia-Antipolis, France

**Keywords:** rhizobium, legumes, symbiosis, nitrogen, photosynthesis, carbon, systemic signaling, plant nutrition

## Abstract

Symbiotic nodules formed on legume roots with rhizobia fix atmospheric N_2_. Bacteria reduce N_2_ to NH_4_^+^ that is assimilated into amino acids by the plant. In return, the plant provides photosynthates to fuel the symbiotic nitrogen fixation. Symbiosis is tightly adjusted to the whole plant nutritional demand and to the plant photosynthetic capacities, but regulatory circuits behind this control remain poorly understood. The use of split-root systems combined with biochemical, physiological, metabolomic, transcriptomic, and genetic approaches revealed that multiple pathways are acting in parallel. Systemic signaling mechanisms of the plant N demand are required for the control of nodule organogenesis, mature nodule functioning, and nodule senescence. N-satiety/N-deficit systemic signaling correlates with rapid variations of the nodules’ sugar levels, tuning symbiosis by C resources allocation. These mechanisms are responsible for the adjustment of plant symbiotic capacities to the mineral N resources. On the one hand, if mineral N can satisfy the plant N demand, nodule formation is inhibited, and nodule senescence is activated. On the other hand, local conditions (abiotic stresses) may impair symbiotic activity resulting in plant N limitation. In these conditions, systemic signaling may compensate the N deficit by stimulating symbiotic root N foraging. In the past decade, several molecular components of the systemic signaling pathways controlling nodule formation have been identified, but a major challenge remains, that is, to understand their specificity as compared to the mechanisms of non-symbiotic plants that control root development and how they contribute to the whole plant phenotypes. Less is known about the control of mature nodule development and functioning by N and C nutritional status of the plant, but a hypothetical model involving the sucrose allocation to the nodule as a systemic signaling process, the oxidative pentose phosphate pathway, and the redox status as potential effectors of this signaling is emerging. This work highlights the importance of organism integration in plant biology.

## Introduction

Nitrate (NO_3_^−^) and ammonium (NH_4_^+^) are the major forms of inorganic nitrogen (N) in the soil. However, plant growth in terrestrial ecosystems is often limited by N availability (Verhoeven et al., 1996; Ågren et al., 2012). Approximately 65 million years ago, plants of the legume family (Fabaceae) and soil bacteria of the Rhizobia type gain the capacity to establish a symbiosis whose function is to reduce atmospheric nitrogen (N_2_) to ammonia (NH_3_/NH_4_^+^) within the bacteria, and then transfer the NH_4_^+^ to the plant when its N demand is not satisfied by mineral N present in the soil (Roy et al., 2020). Although atmospheric N_2_ is a non-limiting N resource, symbiotic nitrogen fixation (SNF) generally does not entirely meet the plant’s N requirements. Indeed, N acquisition through SNF generally does not reach the same level as the uptake of NO_3_^−^ and NH_4_^+^ when these ions are present in non-limiting concentrations (Ruffel et al., 2008). However, symbiosis allows legume holobionts (i.e., plant in association with its symbiotic bacteria) to grow on poor soils lacking inorganic N. Nevertheless, when enough mineral N is present in soils, legume symbiosis is inhibited and plants satisfy their N demand by mineral N acquisition as non-symbiotic higher plants.

**Figure 1:**
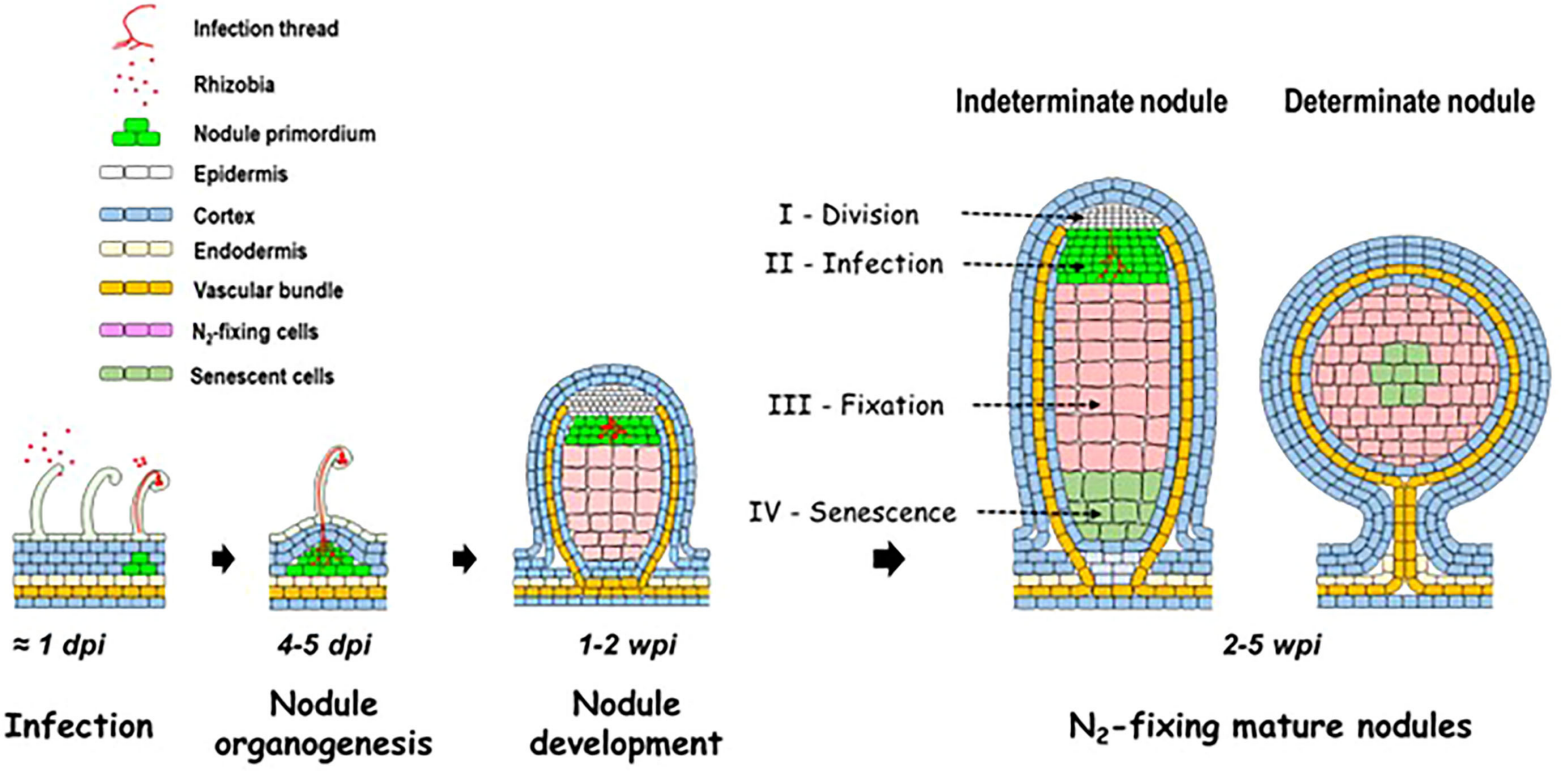
Schematic representation of the establishment and of the development of the legume–rhizobium symbiosis, and of the structure of mature indeterminate and determinate nodules. dpi, day post-inoculation; wpi, week post-inoculation.

**Figure 2:**
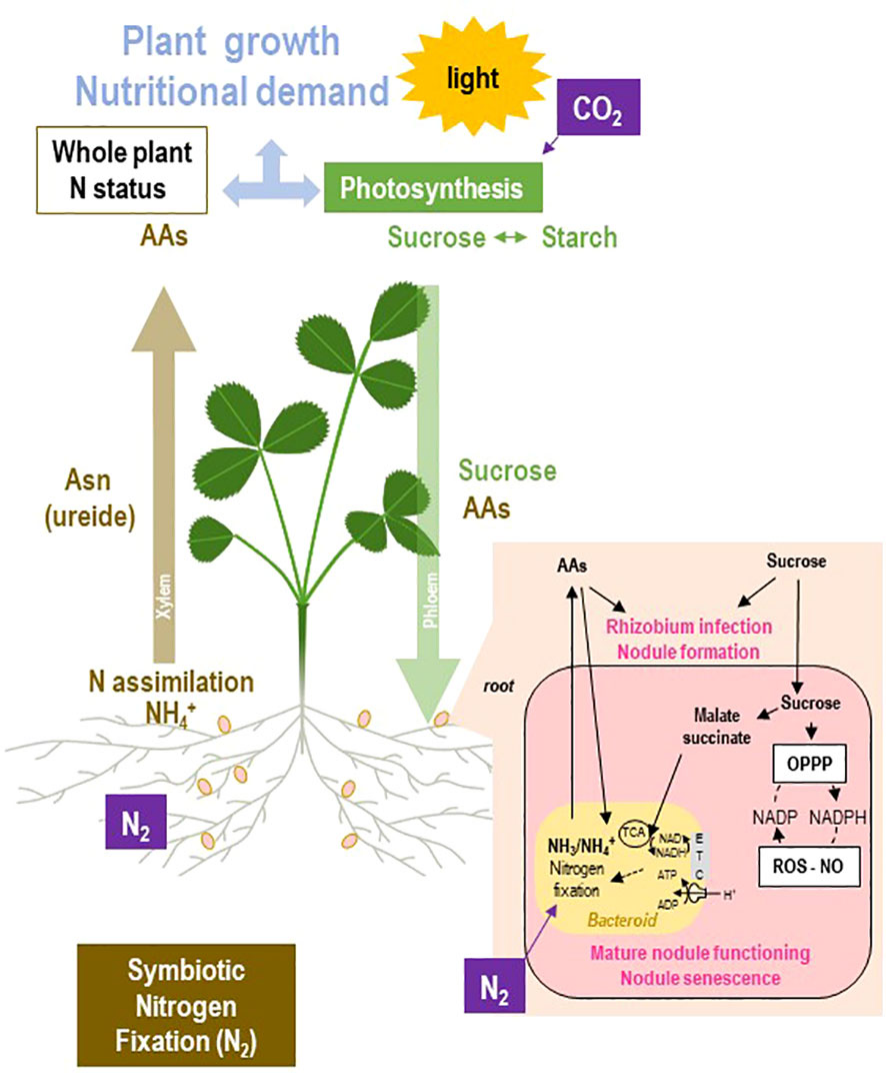
Overview of the N and C fluxes within the rhizobium–legume holobiont using symbiotic nitrogen fixation as the sole source of nitrogen. Atmospheric N_2_ is fixed into NH_3_/NH_4_^+^ by nitrogenase in the bacteroids of the root nodule. The resulting NH_4_^+^ is delivered to the plant cells where it is assimilated into amino acids (AAs). In temperate legumes, asparagine (Asn) is the main form of transport of nitrogen from the root to the shoot through the xylem flux, whereas in some tropical legumes, this transport involves ureides. Photosynthesis allows the plant to capture light and CO_2_ to produce sugars that are either distributed as sucrose within the plant through the phloem to the “sink” organs or stored as starch. Sucrose is delivered to the roots together with AAs enabling nodule development and symbiotic activity. Nitrogen fixation is energy costly and requires intense fueling by the plant to the symbiont. Malate and succinate are the main sources of carbon and energy provided by the plant to the bacteroid, where they are used through the tricarboxylic acid (TCA) cycle to generate reducing power and ATP. Despite the fact that nitrogen fixation produces large amounts of NH_4_^+^, the assimilation of NH_4_^+^ is repressed in bacteroids, and therefore amino acids are acquired by the bacteroid from the plant. Nodule sucrose also fuels the oxidative pentose phosphate pathway (OPPP) regenerating the reducing power required to maintain redox homeostasis under the microoxic conditions of nodules. The plant growth determines the N and C nutritional requirements of the plant. ETC, electron transfer chain; NO, nitric oxide; ROS, reactive oxygen species.

SNF takes place in a new organ, the nodule (**Figure 1**), in which the plant hosts and nourishes the bacteria (Oldroyd et al., 2011). After a stage of mutual recognition of the two partners, involving plant flavonoids and bacterial lipochito-oligosaccharides (the Nod factors), the bacteria penetrate inside the root hairs *via* a specific structure, the infection thread, while the root cortical cells divide to initiate nodule formation (Roy et al., 2020). The infection thread grows and crosses the root hair and then the cortical cells to reach the cells of the young growing nodule inside which the bacteria are released. In the nodule, the bacteria differentiate into bacteroids and acquire the ability to reduce N_2_ to NH_3_/NH_4_^+^ through the activity of a specific enzyme, the nitrogenase (Oldroyd and Downie, 2008). The nodules are of either indeterminate (clover, *Medicago*, alfalfa, and pea) or determinate (soybean, cowpea, and bean) type (Hirsch, 1992). Indeterminate nodules have a persistent meristem and are composed of four distinct zones (**Figure 1**): zone I (meristematic zone) where cells divide, zone II (infection zone) where bacteria infect cells of the plant and differentiate into bacteroids (i.e., specialized terminally differentiated bacteria unable to divide anymore), zone III (fixation zone) where the bacteroids reduce N_2_ to NH_3_/NH_4_^+^, and zone IV (senescence zone) where plant cells and bacteroids enter in senescence (Timmers et al., 2000). The determinate nodules have no persistent meristem and develop by cell expansion. Reduction of N_2_ by nitrogenase and subsequent transfer of NH_4_^+^ to the plant partner is the central process of symbiosis (**Figure 2**; Oldroyd and Downie, 2008). As nitrogenase is irreversibly inhibited by traces of oxygen (O_2_), the concentration of O_2_ inside the nodules is very low, approximately 10 to 40 nM (Appleby, 1992). Thus, the nodules must move from a normoxic environment, at the beginning of their development, to a microoxic one in the fixation zone of mature N_2_-fixing nodules. In exchange for reduced N, the plant provides carbon (C) in the form of dicarboxylic acids to the bacterial partner (Yurgel and Kahn, 2004; Udvardi and Poole, 2013). Terminally differentiated bacteroids display a metabolic specialization in nitrogen fixation. On the one hand, repression of NH_4_^+^ assimilation through the Glutamine synthetase/Glutamate synthase (GS/GOGAT) cycle makes the bacteroids dependent on amino acids supplied by the plant (Patriarca et al., 2002; Prell et al., 2009; Oldroyd et al., 2011). On the other hand, NH_4_^+^ produced by nitrogenase is exported outside of the bacteroid, acquired, and actively assimilated in surrounding plant cells in the presence of carbon skeleton acceptors derived from photosynthates translocated from shoots to roots and nodules (**Figure 2**).

The N supply to the plant by nodules requires various regulatory mechanisms operating through the interplay between C and N metabolisms, the sensing of the whole plant N status, the adjustment of nodule capacity to the plant N demand, the supply of O_2_ to the nodules, and/or the redox homeostasis (**Figure 2**; Schulze, 2004; Schwember et al., 2019; Lindström and Mousavi, 2020; Chaulagain and Frugoli, 2021). Over the last 15 years, multiple regulatory mechanisms, acting either locally in response to the nodule environment or at the whole symbiotic plant level and involving systemic signaling, have been evidenced. This review aims to summarize our current knowledge on the regulation of the N-fixing symbiosis by the plant N and C nutritional status through the interplay of N, C, and energy metabolisms, and the diverse local and systemic signaling mechanisms. The current knowledge of the various pathways characterized at the molecular and genetic levels will be discussed regarding their impact on the whole plant phenotype. Finally, we will focus on the future challenges toward the understanding of the control of symbiosis by the plant nutritional demand and the attempt to understand crosstalk, interplays, and emerging properties of symbiotic holobionts in the context of a fluctuating environment. Oxygen plays a key role in regulating nitrogen fixation and nodule energy metabolism (Schulze, 2004; Schwember et al., 2019; Booth et al., 2021; Schulte et al., 2021). This topic is not exhaustively reviewed in this paper but will be occasionally mentioned when a crosstalk with regulation of symbiosis by the plant nutritional demand is suggested.

## Symbiotic capacities are often limited by the environment or restricted by the plant

Rhizobium–legume symbiosis may be seen as an adaptive response of the legume–rhizobium holobiont to circumvent plant N deficit by activating a new N acquisition pathway from air, an unlimited N source (**Figure 2**). However, legume plants relying on N_2_ fixation are frequently N limited, indicating that symbiosis may not be sufficient to fulfill alone the N requirements for plant optimal nitrogen nutrition (Crozat et al., 1994; Gan et al., 2002; Moreau et al., 2008). Multiple factors contribute to the limitation of symbiotic capacity.

A major cause of this limitation is the carbon cost of the nitrogen fixation process (Minchin and Witty, 2005). It is generally observed that symbiotic development is tightly correlated to the plant capacity to supply the symbiotic structures with carbon (C) metabolites required for its formation, persistence, and functioning (Walsh et al., 1987; Voisin et al., 2003). Although nodules represent a small part of the plant mass, they can consume more than 25% of the products of photosynthesis for SNF (Schuize et al., 1999; Vance, 2008). The carbon cost per unit of fixed N (g C per g N fixed) was shown to vary widely with species, growth stage, and environmental conditions, ranging from 1.4 to 12 g C per gram fixed N (Schwember et al., 2019). The limitation of symbiosis by photosynthesis and the supply of carbon to the nodules has been a matter of debate. On one hand, some authors have argued that under normal growth conditions (non-limiting water and mineral supply, optimal photoperiod, and light intensity), the supply of sugars from photosynthesis to the nodules may not be limiting (Vance and Heichel, 1991; Schulze, 2004). On the other hand, under environmental stress, such as water deficiency, a reduced availability of C for bacteroid respiration and nitrogenase activity was associated with the decline in N_2_ fixation (Gordon et al., 1999; Baier et al., 2007). Furthermore, several studies have shown that elevated CO_2_ concentrations (eCO_2_) stimulate N_2_ fixation and plant biomass production, demonstrating that photosynthesis is effectively limiting symbiosis (Rogers et al., 2006; Sanz-Sáez et al., 2010; Lam et al., 2012; Li et al., 2017; Parvin et al., 2020). In *M. truncatula* under eCO_2_, nodule number and size are increased and most N_2_ fixation-related genes are upregulated (Guo et al., 2013). This was further confirmed by Parvin et al. (2020) who showed, either in normal growth condition or under hydric stress, that faba bean under eCO_2_ is only able to increase its C gain if nodule activity is maintained. This response of symbiotic legumes to eCO_2_ is original as compared to non-symbiotic C3 plants supplied by NO_3_^−^ as N source displaying an eCO_2_ acclimation and a reduction of NO_3_^−^ uptake and assimilation (Stitt and Krapp, 1999; Rogers et al., 2006; Guo et al., 2013). The causes of this acclimation to eCO_2_ are not yet understood, but several hypotheses have been recently raised, such as a lower NO_3_^−^ concentration in most plant organs, a reduced NO_3_^−^ acquisition due to a decreased leaf transpiration, an insufficient NADH power for NO_3_^−^ reduction due to reduced photorespiration under eCO_2_, or the repression of most NO_3_^−^ uptake and assimilation systems by eCO_2_ (Gojon et al., 2022).

**Figure 3:**
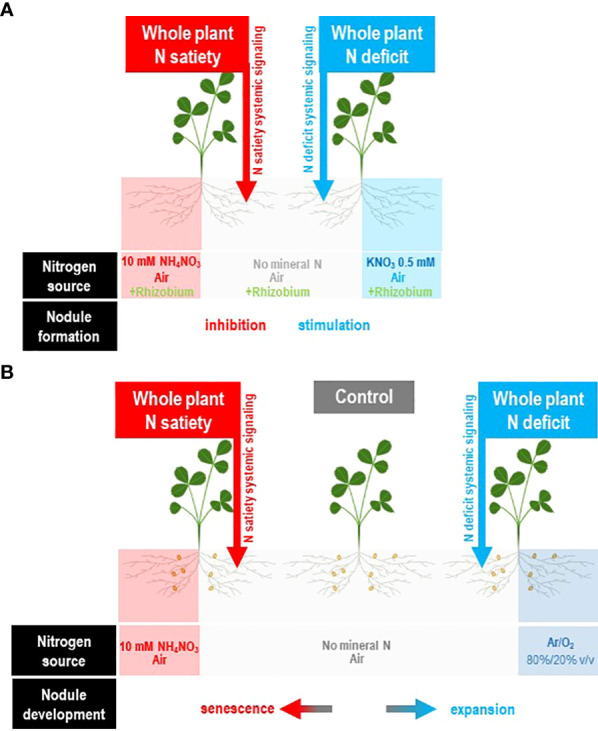
Split-root systems to characterize the control of nitrogen-fixing symbiosis by systemic N signaling of the plant N demand. The experimental systems are described in the original studies of Ruffel et al. (2008); Jeudy et al. (2010); Laguerre et al. (2012); Lambert et al. (2020), and Pervent et al. (2021). *M. truncatula* plants are cultivated hydroponically. The root system of each plant is separated into two compartments. Each compartment receives specific N supplies: either low level of mineral N supply (0.5 mM KNO_3_), high level of mineral N supply, air (N_2_/O_2_ 80/20 v/v), or no gaseous N supply (Ar/O_2_ 80/20 v/v). At the beginning of the experiment, a contrasted N supply is applied to a half-root system. The N treatment modifies the whole plant N status and the whole plant N demand without affecting the environment and the untreated half-root system. Response of the untreated roots to this treatment results necessarily from systemic signaling originated from the rest of plant to these roots. **(A)** Split root systems used to study the control of nodule formation by plant N demand (Pervent et al., 2021). Non symbiotic plants are used. Contrasted mineral N supply applied to half-root systems may either fully satisfy the plant N demand (N satiety) or results in plant N limitation (N deficit). The response to inoculation by rhizobium in untreated roots strongly relies on the level of N supply of the plant revealing the strong systemic control of nodule formation by systemic signaling of the plant N demand. **(B)** Split root systems used to study the control of mature nodules by the plant N demand (Jeudy et al., 2010; Laguerre et al., 2012; Lambert et al., 2020). Symbiotic plants supplied with air as a unique N source through the function of mature N_2_ fixing nodules are used. N treatment applied to a half-root system may either fully satisfy the plant N demand (N satiety) or result in plant N limitation (N deficit). Plant N satiety resulted in nodule senescence whereas plant N limitation stimulates the expansion of mature nodules. The development of the nodules in untreated roots is strongly dependent on the level of supply of the whole plant, revealing the control by the systemic signaling of the whole plant N demand.

Nevertheless, C supply is not the only cause of the limitation of symbiotic capacities. Firstly, the nodule development process in N-limited plants requires several days to result to active SNF. Severe N deficit during this lag period is often detrimental for plant growth (especially if the N demand of the young plant is high) and may inhibit the process (Moreau et al., 2008). Secondly, as soon as the symbiosis is established, the SNF efficiency is frequently not at its maximum. Compatible rhizobia forming natural populations in the soil, able to form nodule with a legume host, may result in contrasted levels of SNF (Laguerre et al., 2012; Bourion et al., 2017; Boivin et al., 2020). Thirdly, symbiotic organs are highly sensitive to local environmental abiotic constraints such as drought, heavy metal, temperature, soil pH, or mineral deficiencies (phosphorus, sulfur) that may drastically inhibit SNF (Durand et al., 1987; Liu et al., 2011; Ferguson et al., 2013; Marino et al., 2013; Gil-Quintana et al., 2013b). The dynamics and the fluctuation of these constraints, as well as the time required to establish the new symbiotic structures necessary for the plant N limitation recovery, must also be considered. Fourthly, the nodule proliferation is tightly controlled and generally limited by the plant at multiple steps of the nodule development. Split-root studies have been used to characterize the systemic control of symbiosis by the whole plant (**Figure 3**). The autoregulation of nodule number (AON) mechanism limits the new infections by compatible rhizobia as soon as a first infection wave is progressing toward the formation of the symbiotic organ (Kosslak et al., 1983; Kosslak and Bohlool, 1984; Mathews et al., 1989; Olsson et al., 1989; Reid et al., 2011b; Kassaw et al., 2015; Ferguson et al., 2019). The inhibition occurs rapidly after infection before the completion of organogenesis and the activation of nitrogen fixation activity. The formation of spontaneous pseudo-nodules or nodules induced by *R. meliloti* mutants defective in their ability to invade and multiply within host tissues elicits the AON-related feedback suppression of nodule formation similarly to that elicited by the wild-type bacteria (Caetano-Anollés et al., 1990; Caetano-Anollés et al., 1991). Therefore, AON is related to developmental rather than to nutritional feedback. However, AON is also regulated by the plant N demand. In response to N deficit, the symbiotic plant releases the AON repression to increase its nodule number (**Figure 3A**; Jeudy et al., 2010; Laguerre et al., 2012). Fifthly, when the symbiotic organ is formed, its behavior remained tightly controlled by the plant. At the nodule level, the absence of N_2_ fixation triggers a local “plant sanction” response associated with the rapid arrest of nodule growth and the reduction of cultivable bacteria in the nodule (Kiers et al., 2003; Oono et al., 2009; Oono et al., 2011). Mature nodule development is tightly controlled by the systemic signaling of the plant nitrogen demand especially in indeterminate legumes (**Figure 3B**). On one hand, the plant N deficit stimulates the nodule expansion (as well as bacteroid differentiation), resulting in an increase in N_2_ fixation activity (Jeudy et al., 2010; Laguerre et al., 2012). On the other hand, the plant N satiety activates the destruction of bacteroids and the senescence of the organ (Pérez Guerra et al., 2010; Lambert et al., 2020).

## Symbiotic development and function are tightly correlated to carbon allocation from the shoot to the symbiotic organs

**Figure 4:**
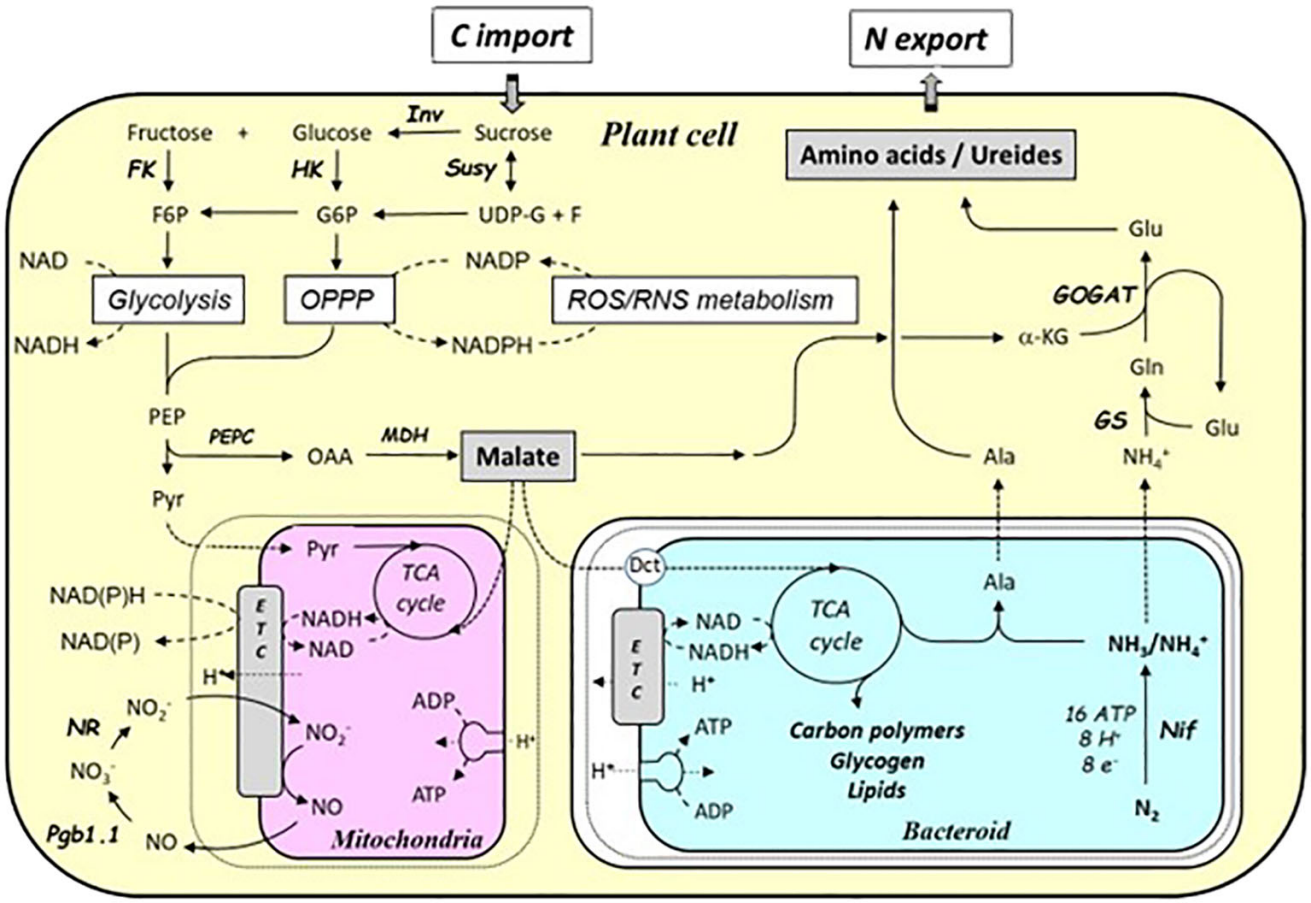
Schematic representation of the carbon and nitrogen metabolic pathways in mature nodules. Sucrose is first oxidized through glycolysis and oxidative pentose phosphate pathway to generate reducing power (NADH, NADPH) and phospho*enol*pyruvate (PEP). PEP, on the one hand, supplies pyruvate to the mitochondria to fuel energy metabolism and, on the other hand, supplies the carbon skeletons to produce organic acids (malate, succinate). Part of the organic acids are supplied to the bacteroids and contribute to generating the reducing power and the energy necessary for the reduction of atmospheric nitrogen (N_2_) into ammonia (NH_4_^+^) by nitrogenase. Another part is used to produce the α-keto acids involved in the assimilation of NH_4_^+^ from bacteroids, *via* the glutamine synthetase/glutamate synthase pathway. Assimilated N is exported, as either amino acids (indetermined nodules) or ureides (determinate nodules), to the whole plant. Ala, alanine; Dct, dicarboxylate transporter; e^-^, electron; ETC, electron transfer chain; F6P, fructose-6-phosphate; FK, fructokinase; G6P, glucose-6-phosphate; GOGAT, glutamate synthase; Gln, glutamine; Glu, glutamate; GS, glutamine synthetase; H^+^, proton; HK, hexokinase; Inv, invertase; α-KG, α-ketoglutarate; MDH, malate dehydrogenase; N_2_, dinitrogen; NH_4_^+^, ammonium; Nif, nitrogenase; NO, nitric oxide; NO_2_^−^, nitrite; NO_3_^−^, nitrate; NR, nitrate reductase; OPPP, oxidative pentose phosphate pathway; PEP, phospho*enol*pyruvate, PEPC, PEPC carboxylase; Pgb1.1, phytoglobin 1.1; Pyr, pyruvate; ROS/RNS, reactive oxygen species/reactive nitrogen species; Susy, sucrose synthase; TCA, tricarboxylic acid cycle; UDP-G, UDP glucose.

Several ^14^CO_2_ pulse-chase studies have shown that sucrose from photosynthesis is delivered to the nodules *via* the phloem and then degraded in the cytosol of plant cells to produce organic acids (Gordon et al., 1985; Streeter and Wong, 1988; Rosendahl et al., 1990; **Figure 4**). Sucrose is first metabolized to UDP-glucose plus fructose, *via* sucrose synthase (SS), and then oxidized through glycolysis to phosphoenolpyruvate (PEP). PEP is then successively metabolized to oxaloacetate (OAA) and malate by PEP carboxylase (PEPC) and malate dehydrogenase (MDH), respectively. In the different types of nodules, determined or undetermined, the transport of sucrose and/or organic acids to infected cells is preferentially symplastic or apoplastic, or a combination of both (Booth et al., 2021). Thus, the presence of plasmodesmata in soybean (Brown et al., 1995), faba bean (Abd-Alla et al., 2000), and *Medicago truncatula* (Gaudioso-Pedraza et al., 2018) suggests symplastic transport of C metabolites. Furthermore, the high expression of SWEET-type transporters in vascular parenchyma cells of *M. truncatula* or *Lotus japonicus* (Kryvoruchko et al., 2016; Sugiyama et al., 2017) and ALMT (Takanashi et al., 2016) suggests that there is also an apoplastic pathway for the delivery of either sucrose or organic acids to infected cells. Analysis of the expression and activity of enzymes involved in the conversion of sugars to organic acids shows that both vascular parenchyma, non-infected and infected cells are involved in the process of degrading sucrose to malate, albeit in different ways depending on the nodule type (Vance, 2008; Booth et al., 2021). In the determinate nodules of soybean and chickpea, most of the carbon metabolism occurs in non-infected and vascular parenchyma cells (Kouchi et al., 1988; Day and Copeland, 1991), whereas in indeterminate nodules, such as in pea and *M. sativa*, or in *L. japonicus*, it is more evenly distributed between infected and non-infected cells and vascular parenchyma cells (Fedorova et al., 1999; Hohnjec et al., 2003; Takanashi et al., 2012). Microoxic conditions associated with O_2_ channeling by leghemoglobins prevail in central cells of the nodule as nitrogenase requires a very low O_2_ level to be active. In infected cells, some of the malate is transported to the mitochondria where it is used to regenerate ATP and produce carbon skeletons needed for the assimilation of the reduced nitrogen, i.e., NH_4_^+^, produced in the bacteroids (Gordon et al., 1985; Rosendahl et al., 1990; Smith et al., 2002). Nodule mitochondria are characterized by their ability to produce ATP more efficiently and at lower O_2_ levels than mitochondria in roots and other tissues (Booth et al., 2021). Furthermore, the operation of a phytoglobin-nitric oxide (Pgb-NO) respiration pathway, in which O_2_ is consumed by phytoglobins whose affinity for oxygen (*K*_d_ O_2_ ≈ 2–10 nM) is significantly higher than that of cytochrome oxidase (*K*_d_ O_2_ ≈ 50–200 nM), allows the innermost cells of nodules to regenerate ATP under the microoxic conditions prevailing in nodules (Berger et al., 2019; Berger et al., 2021). The question particularly arises as to what the O_2_ concentration is and what type of respiration is functioning in the infected and uninfected cells in the fixation zone. Indeed, measurements with microelectrodes in determinate nodules such as soybean (Tjepkema and Yocum, 1974) and *L. japonicus* (Denison, 1992), as well as in indeterminate nodules of *M. sativa* (Soupène et al., 1995), revealed a strong O_2_ gradient between the outside and inside of the nodules. However, to our knowledge, there is no direct way to investigate the difference in O_2_ concentration between infected and uninfected cells *in vivo*. Considering that leghemoglobins are exclusively localized in infected cells (Robertson et al., 1984), an O_2_ gradient between infected and uninfected cells could be hypothesized. O_2_ concentration in infected cells has been indirectly calculated by the fractional oxygenation of leghemoglobin (Denison and Layzell, 1991). In addition, several modeling studies have reported that pO_2_ ranges from 12 to 25 µm in the gas spaces surrounding infected and uninfected cells to 10–60 nM in infected cells (Thumfort et al., 1994; Thumfort et al., 1999; Thumfort et al., 2000). Some studies have indirectly addressed the issue of pO_2_ differences between infected and uninfected cells in alfalfa (Arrese-Igor et al., 1993), cowpea (Dakora and Atkins, 1990), or soybean (James et al., 1991) and led to the same conclusion of an O_2_ gradient within the fixation zone. Thus, it is very likely that depending on the local O_2_ concentration, the involvement of Pgb-NO respiration, alongside classical O_2_-dependent respiration, might be important for maintaining the energy state and the metabolism of cells (Berger et al., 2019; Berger et al., 2021). Mitochondria also have high MDH activity and low malic enzyme (ME) activity (Day and Mannix, 1988; Bryce and Day, 1990), which favors the reduction of malate to OAA for subsequent ammonia assimilation (**Figure 4**). Another part of the organic acids is transported in the bacteroids as dicarboxylate, mainly malate (**Figure 4**; Booth et al., 2021). The activity of the dicarboxylate transporter on the symbiosome membrane has been demonstrated, but the protein has not yet been identified. In contrast, the bacteroid membrane transporter, DctA, has been well characterized and identified in *Rhizobium leguminosarum* and *Bradyrhizobium japonicum* (Ronson et al., 1984; Pessi et al., 2007). DctA is upregulated and accounts for most of the carbon influx into the symbiosomes under symbiotic conditions. Once inside the bacteroid, malate is metabolized by the malic enzyme (ME) and MDH to produce pyruvate and OAA, respectively, which fuel the energy and carbon metabolism of the bacteroids (production of ATP and reducing power, storage of excess carbon in the form of carbon polymers, glycogen, and lipids), and allows the reduction of N_2_ to NH_3_ by nitrogenase (Lodwig et al., 2005; Terpolilli et al., 2016; Liu et al., 2018). Nitrogenase is the major bacteroid process of ATP and reducing power consumption (16 ATP and 8 e- per N_2_ fixed; **Figure 4**). A comprehensive study, combining experimental and metabolic modeling approaches, was recently conducted in *R. leguminosarum* and *Azorhizobium caulinodans* to explain the fundamental features of bacteroid metabolism (Schulte et al., 2021). The catabolism of dicarboxylates provides energy for N reduction and allows the synthesis of carbon polymers and alanine. Metabolic modeling of TCA cycle in the bacteroid suggests that catabolism of dicarboxylates induces a higher NADH/NAD ratio than it might have been if fueled by sugars (Schulte et al., 2021). In this study, the authors show that the entire process is dependent on the O_2_ concentration, the low O_2_ content of which protects nitrogenase from inhibition, reduces the assimilation of NH_4_^+^ into glutamate in the bacteroids, and promotes the export of NH_4_^+^ and alanine to the plant cell (Schulte et al., 2021). NH_4_^+^ exported by the bacteroid is transported into the cytosol of the infected host cells for its assimilation in amino acids, *via* the combined action of GS/GOGAT (**Figure 4**; Cordoba et al., 2003; Seabra et al., 2012). This assimilation process requires an additional flux of C skeleton (α-ketoglutarate) provided by photosynthesis to match the NH_4_^+^ flux. Assimilated N is then exported from the nodules to the leaves either as amino acids (mainly asparagine) in indeterminate nodules or as ureides in some determinate nodules (Vance, 2008; Sprent, 2009; Liu et al., 2018). The functioning of the SNF results in a combined action of C and N metabolisms, associated with a strong increase in the expression and activity of their primary enzymes as compared to roots (Vance, 2008), emphasizing the interrelation and co-regulation of the two metabolisms.

## The control of symbiosis by plant N demand involves systemic signaling

Mineral nitrogen and particularly NO_3_^−^ is known to control its root acquisition at multiple levels. These controls may be related to the ion itself or to the product of its assimilation (Ruffel et al., 2008; Ruffel et al., 2014). These controls are exerted at the level of the organ in presence of the ions and involve local signaling, or at the level of the whole plant and involves systemic signaling. Split-root systems have been used to discriminate between these local and systemic signaling (**Figure 3**). NO_3_^−^ itself stimulates root development and induces root activities involved in its uptake and assimilation. It is a common feature of many NO_3_^−^ transporters and enzymes involved in NO_3_^−^ assimilation to be activated by the presence of the ion (Vidal et al., 2020). Root NO_3_^−^ acquisition induction generally requires the presence of the ion at the site of response (local effect) and may occur in absence of assimilation of the ion (Zhang et al., 1999; Wang et al., 2004). At the whole plant level, the main nitrogen sources (NO_3_^−^, NH_4_^+^, N_2_, or amino acids) are finally assimilated into the same downstream N metabolites to fulfill the N requirement generated by plant growth and functioning. The concept of N demand refers to the balance between the N requirement to fulfill the plant growth potential and plant N acquisition capacity (Imsande and Touraine, 1994; Gojon et al., 2009). Plant N demand may be contrasted according to plant growth rates and/or plant N regimes. Plant N satiety is reached when there is full satisfaction of the N demand (excess of mineral nitrogen for example), whereas plant N deficit occurs when the N demand is not fully satisfied, and the N provision limits plant growth. Variation of the level of downstream N metabolites (namely, amino acids) of the shoots translocated in roots may be associated with this variation of N demand, consistent with the hypothesis of amino acids cycling through the phloem being a signal of plant N demand (Imsande, 1986; Muller and Touraine, 1992; Parsons et al., 1993; Tillard et al., 1998; Girin et al., 2010). The control of NO_3_^−^ uptake by a systemic signaling of the plant N demand has been evidenced in several biological systems (Gansel et al., 2001; Girin et al., 2007; Ruffel et al., 2008). N-satiety signaling represses NO_3_^−^ transporters, while N-deficit signaling upregulates them. Evidence of regulation of root development by similar systemic control has been also evidenced (Forde, 2002). Although mechanisms behind local and systemic N signaling can be discriminated, they share many targets and, at the whole plant level, generally coexist and interact actively. Without specific experimental designs such as plant cultivated in split-root systems, it is therefore difficult to discriminate between these two modes (**Figure 3**).

There is little evidence on the impact of the plant N status on the early interactions between rhizobium and legume roots (Grillo et al., 2016). However, following the early interaction, the development of symbiosis requires a whole plant N deficit and is suppressed when plant is supplied by high mineral N supply (Streeter and Wong, 1988; Pervent et al., 2021). However, the plant N deficit must not be too extreme because when the seed reserves are totally consumed, nitrogen and carbon metabolites are still needed to form the new symbiotic structures. This argues for the empiric practice used by legume growers of adding a little amount of mineral nitrogen fertilizer as a “starter” at sowing before symbiosis establishment (Imsande, 1986; Streeter and Wong, 1988). Nevertheless, if seed reserves are consumed and if the plant is not able to fulfill its N demand with the mineral N, active symbiosis may be established. The use of split-root systems demonstrated that the control of nodulation by N demand is mainly exerted at the whole plant level (**Figure 3A**). Nodule formation requires whole plant N-deficit systemic signaling (Streeter and Wong, 1988; Pervent et al., 2021). Split-root studies in *M. truncatula* did not argue for a strong local effect of NO_3_^−^ itself on nodule formation (Pervent et al., 2021), suggesting that repression of nodulation by NO_3_^−^ is mainly related to the downstream N-metabolite production in the whole plant rather than to its presence in the nodule environment. The responses of *M. truncatula* root to systemic N signaling during the interaction with rhizobium and the nodule formation process were characterized (Pervent et al., 2021). The accumulation of many transcripts associated with the transcriptome reprogramming in response to rhizobium requires systemic signaling of N deficit and is repressed by systemic N signaling of N satiety. However, it is likely that systemic N signaling tunes the progression of the process rather than determine a capacity of the plant to respond to rhizobium (Pervent et al., 2021). Globally, the impact of systemic N signaling is more pronounced during nodule organogenesis, bacteroid differentiation, and activation of nitrogen fixation than on early phases of the interaction.

Evidence for a local regulation of mature symbiotic organs by the efficiency of SNF has been reported. Suppressing nitrogen fixation by Ar/O_2_ treatments in split-root systems results locally in a rapid inhibition of nodule growth (Singleton and van Kessel, 1987; Kiers et al., 2003; Jeudy et al., 2010). The general small size and the early developmental arrest of nodules formed by fix− bacteria are globally in agreement with an inhibition of nodule development in the absence of N_2_ fixation (Laguerre et al., 2012). It was proposed that the plant develops a local nodule autonomous mechanism to restrict the development of nodules formed with ineffective bacteria (Kiers et al., 2003; Oono et al., 2011). Long-term Ar/O_2_ treatments of determinate nodules resulted in a decrease of bacteroid fitness in nodules, associated with early nodule senescence (Kiers et al., 2003; Oono et al., 2009). This result was interpreted as a “host sanction” toward the less beneficial partners. In an evolutionary point of view, “sanction” tends to limit ineffective rhizobia multiplication and dispersion when they are released in the soil. The concept has been extended to indeterminate nodules although bacteroids are terminally differentiated and only undifferentiated bacteria are able to multiply (Oono and Denison, 2010; Oono et al., 2011). Although several reports in soybean/*Bradyrhizobium* and *Medicago/Sinorhizobium* symbioses indicate that mutations suppressing N_2_ fixation do not necessarily impact the reproductive fitness of rhizobia present in nodule (Marco et al., 2009; Laguerre et al., 2012), evidence for a long-term stimulation of the reproductive fitness of fix^+^ rhizobia versus fix− rhizobia in co-infected root system of *Mimosa pudica*/*Cupriavidus taiwanensis* have been also reported (Daubech et al., 2017). Mechanisms related to nodule oxygen permeability or pH have been proposed to be instrumental in the local control of legume–rhizobium symbiotic organs (Hunt and Layzell, 1993; Kiers et al., 2003) but, to date, they remain to be precisely elucidated: whether they are the cause, or the consequence of nitrogen fixation inhibition remains unknown.

**Figure 5:**
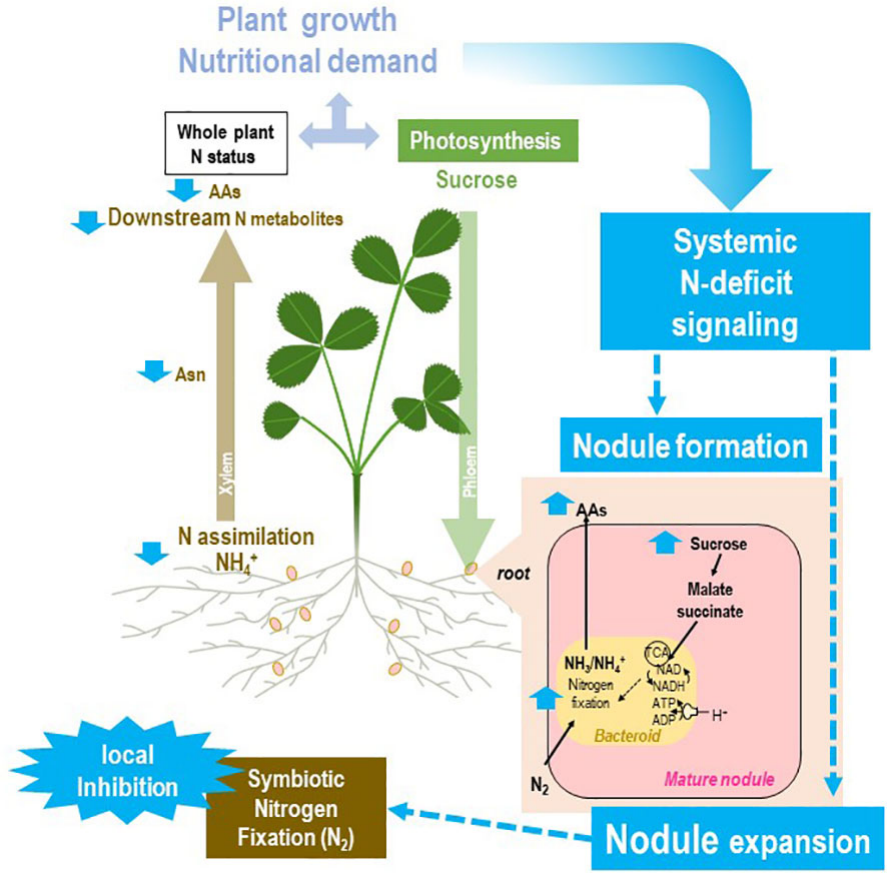
Systemic responses of the rhizobium–legume holobiont to plant N deficit. The general framework of the figure is described in **Figure 2**. Split-root systems used to characterize N-demand signaling are described in **Figure 3**. Various steps of the regulatory loop are indicated in blue. A local suppression of SNF may be obtained artificially (by replacing locally air by a mixture Ar/O_2_ 80/20 v/v) or as the result of abiotic stresses. The local inhibition of symbiosis in the roots exposed to these conditions results in a partial decrease of the whole plant SNF. As the whole plant N demand is not fully satisfied, the systemic signaling promoting symbiosis is activated, resulting in the formation of new nodules on the other roots not exposed to the constraint. In mature nodules of these roots, the N-deficit systemic signaling results in a strong increase in nodule sucrose and organic acid levels associated with nodule expansion. This increase in nodule biomass is associated with higher levels of SNF in roots not exposed to the local constraint that may compensate the plant N deficit.

**Figure 6:**
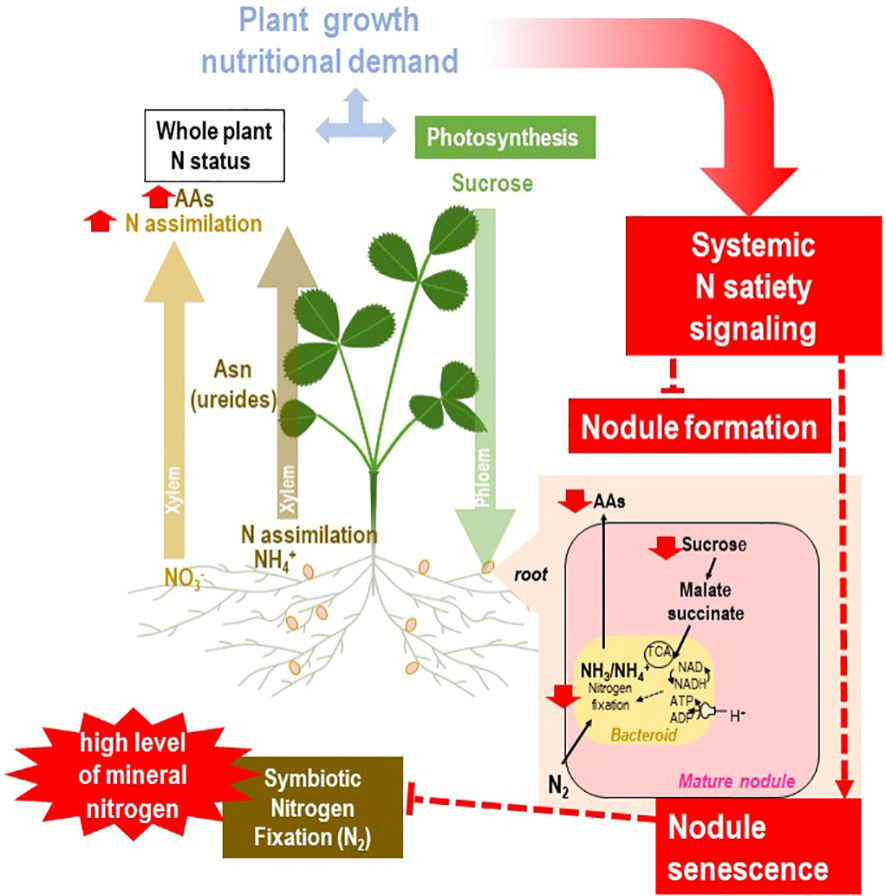
Systemic responses of the rhizobium–legume holobiont to plant N satiety. The general framework of the figure is described in **Figure 2**. Split-root systems used to characterize N-demand signaling are described in **Figure 3**. Various steps of the regulatory loop are indicated in red. The local availability of sufficient resources of mineral nitrogen fulfills the plant N demand. In agricultural aerated soils, NO_3_^−^ is generally the main nitrogen source of annual crops. Assimilation of NO_3_^−^ occurs mainly in shoots. Satisfaction of the whole N demand results in repressive systemic signaling, arresting nodule formation. In mature nodules, the N-satiety systemic signaling results in drastic reduction of nodule sucrose levels and rapid activation of nodule senescence and bacteroid proteolysis, associated with a sharp decrease in nitrogen fixation.

Mature nodules are also under the control of systemic signaling of the whole plant N demand (**Figures 3**, **5**, **6**). SNF is highly sensitive to abiotic stress that may locally suppress plant N acquisition capacity (Durand et al., 1987; Marino et al., 2013; Gil-Quintana et al., 2013a), resulting in whole plant N deficit (**Figure 5**). Local suppression of N_2_ fixation in split-root systems by Ar/O_2_ treatment or by inoculation with fix− bacteria of a fix− half-root system results in a compensatory response on the remaining fix+ half-root system (**Figures 3B**, **5**; Jeudy et al., 2010; Laguerre et al., 2012). The stimulation of mature nodule expansion and the formation of new symbiotic organs are observed (Jeudy et al., 2010; Laguerre et al., 2012), both tending to increase the nitrogen fixation of the fix+ half-root system (**Figures 3B**, **5**). This is of biological importance as plants are sessile organisms; they face soil conditions highly variable in time and space. This systemic mechanism contributes to adjust the root N_2_ fixation capacity to the whole plant N demand and to symbiotic root “foraging”. However, it operates probably at the root bundle rather than at the nodule level (Laguerre et al., 2012). Plant facing a uniform reduction of its symbiotic root capacity (fix+ and fix− nodules uniformly distributed on the root system) is unable to trigger the systemic response probably because the plant cannot discriminate between efficient and inefficient root bundles and allocate resources preferentially to the efficient ones (Laguerre et al., 2012). The counterpart of the systemic stimulation of symbiosis by plant N deficit is its systemic repression by plant N satiety (**Figures 3**, **6**). The supply of high level of mineral to a half-root system of N_2_ fixing plants cultivated in a split-root system results in systemic N-satiety signaling, represses nitrogen fixation, and activates the senescence of the nodules and the degradation of nitrogen-fixing bacteroids (**Figures 3**, **6**; Pérez Guerra et al., 2010; Lambert et al., 2020). A control of symbiotic activity by downstream N metabolites produced in the shoots and translocated to the roots by the phloem has been frequently suggested (Parsons et al., 1993; Imsande and Touraine, 1994; Bacanamwo and Harper, 1997; Neo and Layzell, 1997). Amino acid supply has indeed a strong inhibitory effect on symbiosis (Bacanamwo and Harper, 1997). However, because amino acids may also be a source of nitrogen metabolized by the plant roots, additional evidence is required to confirm this “feedback” model. Transcriptome analysis of *M. truncatula* plants cultivated in split-root systems revealed that N-demand systemic signaling is a major driver of nodule development and functioning (**Figures 5**, **6**; Lambert et al., 2020). Although plant N satiety activates a general bacterial transcript breakdown associated with bacteroid lysis during nodule senescence, there is little evidence of a gene-specific regulatory effect of systemic signaling of plant N demand on bacteroid transcriptome. However, plant N satiety and plant N-deficit systemic signaling respectively activates and downregulates numerous plant transcripts involved in nodule senescence, while respectively inhibiting and activating transcript families involved in bacteroid differentiation, nodule meristematic cell division, leghemoglobin synthesis, and sucrose transport (Lambert et al., 2020). They are also associated with large reprogramming of hormonal and plant defense genes (Lambert et al., 2020).

## Mechanisms underlying the regulation of symbiosis by photosynthesis

**Figure 7:**
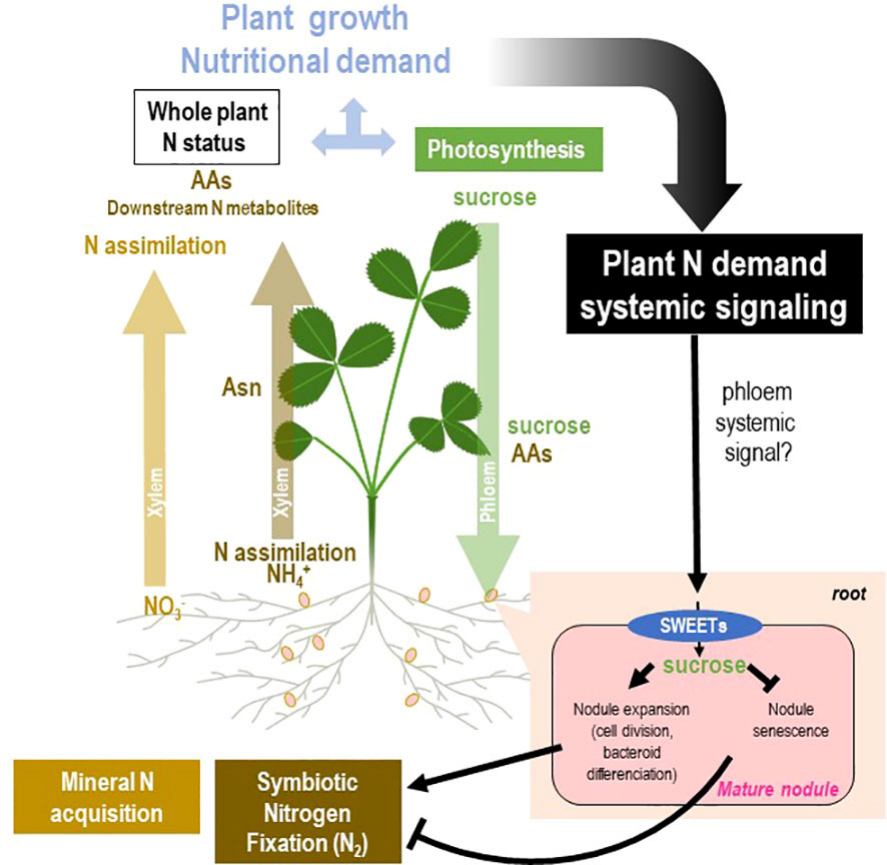
Model of systemic regulation of mature nodules by N demand through nodule sucrose allocation. The general framework of the figure is described in **Figure 2**. Split-root systems used to characterize N-demand signaling are described in **Figure 3**. Systemic control of mature nodule development and activity by the whole plant N demand is associated with variation of the allocation of sucrose produced by the photosynthesis to the mature nodule. The hypothetical systemic signal(s) translocated through the phloem remains unknown. On the one hand, the systemic N-satiety signaling lowers nodule sucrose levels and triggers nodule senescence. On the other hand, the systemic N-deficit signaling increases nodule sucrose levels and stimulates nodule expansion. This differential sucrose allocation associates with variations of the SWEET sucrose efflux transporter transcript levels in response to N-satiety and N-deficit systemic signaling.

Active nodules require a large flux of sucrose from the plant to fuel the N_2_ fixation in the bacteroids (energy and reducing power) and to assimilate the NH_4_^+^ released in plant cells. Despite numerous physiological evidence highlighting the tight coordination of photosynthesis and symbiotic activity, mechanisms responsible for the control of symbiosis by the plant C status remain unknown at the genetic and/or molecular levels. Both N-deficit and N-satiety signaling were associated with rapid variations of sucrose allocation from the plant to the active nodule (Lambert et al., 2020). This suggested the hypothesis that plant sucrose allocation is a systemic signal that modulates nodule activity as a function of plant N demand (**Figure 7**). Consistently with this model, sucrose, produced by shoot photosynthesis, is a major metabolite of phloem sap and its flux is expected to be correlated to the plant growth capacity. Interestingly, nodule Sweet sucrose transporters transcripts have been identified as potential targets of both N-satiety and N-deficit systemic signaling, supporting this hypothesis (Lambert et al., 2020). However, whether sucrose allocation variation is a signal by itself or the consequence of another signaling mechanism remains unknown. The central role of nodule sucrose in the control of symbiosis was already suggested by earlier studies in N-limited supply conditions and in response to drought (Baier et al., 2007). The drop in SNF in response to water stress correlated in several grain legume species (soybean, pea, and bean) to the rapid decline of Susy activity leading to sugar accumulation and organic acid depletion in the nodules (González et al., 1995; González et al., 1998; Gordon et al., 1999; Gálvez et al., 2005). This suggested a key role for Susy in the regulation of SNF by carbon. However, in *M. sativa* and *M. truncatula*, the drop in Susy activity only occurs after inhibition of SNF, which questions the possible role of the Susy in this regulation in forage legumes (Naya et al., 2007; Larrainzar et al., 2009).

**Figure 8:**
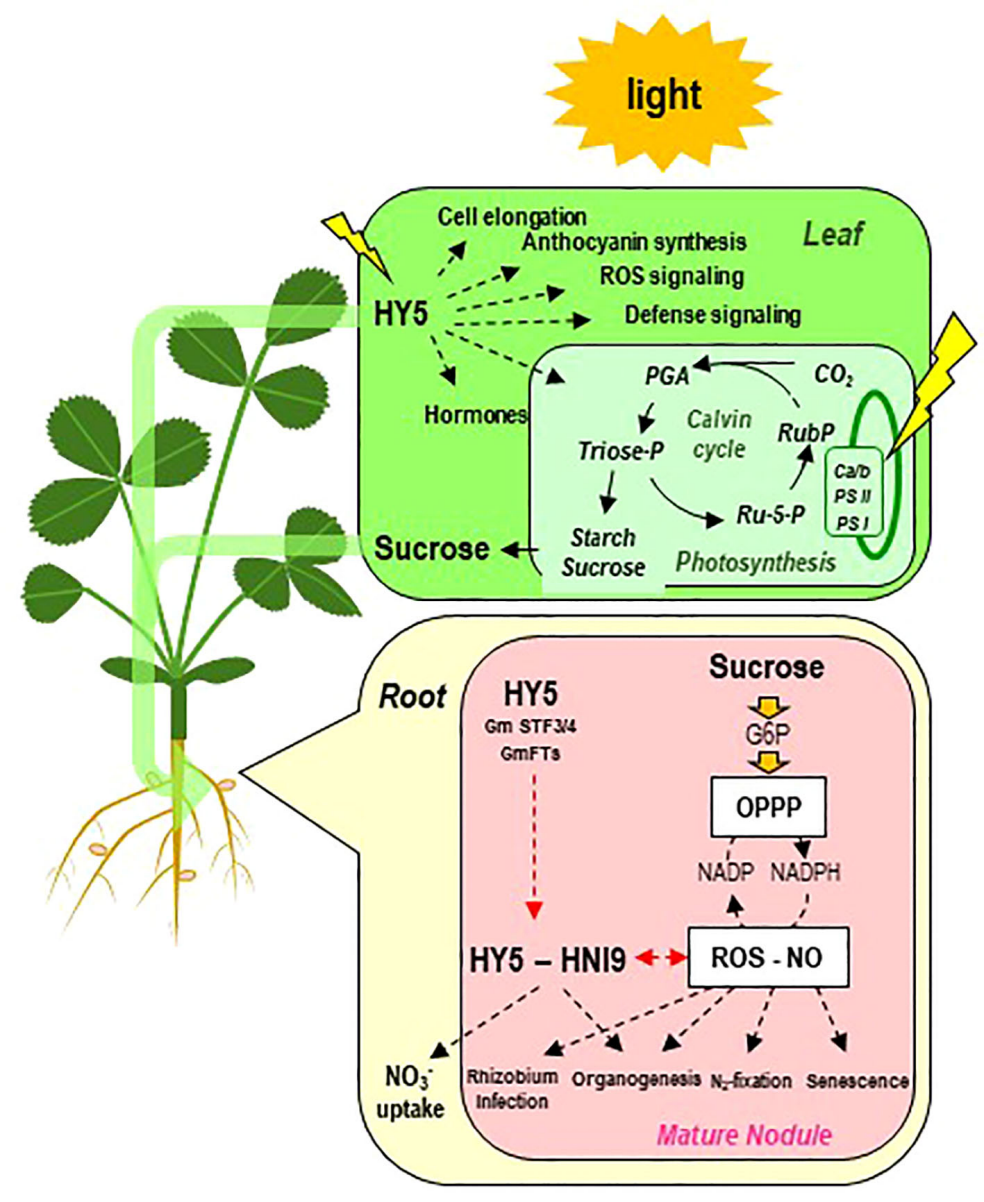
Schematic overview of the current knowledge of regulatory pathways potentially involved in the control of symbiosis by photosynthesis. On the one hand, sucrose resulting from photosynthesis is exported to the roots and the nodules. The fraction of sugars metabolized *via* the oxidative pentose phosphate pathway (OPPP) generates reducing power (*via* the NADP/NADPH ratio), controlling the cellular redox state (ROS-NO). Redox state is involved in the regulation of many aspects of the establishment and the functioning of symbiosis. In the root, the redox state and the OPPP are also implicated in the regulation of NO_3_^−^ transporters. On the other hand, in the leaves, the b-ZIP transcription factor ELONGATED HYPOCOTHYL 5 (HY5) is activated by light and regulates the assimilation and export of carbon to the root system. HY5 may translocate from shoots to roots *via* the phloem. In roots, HY5 together with the nuclear factor HIGH NITROGEN INSENSITIVE 9 (HNI9) activates the ROS detoxification program in connection with the cellular redox state and regulates downstream NO_3_^−^ transporters and nodule organogenesis (in soybean). Ca/b, chlorophyll a/b binding complex; PS, photosystem; G6P, glucose-6-phosphate; NO, nitric oxide; OPPP, oxidative pentose phosphate pathway; PGA, 3-phosphoglycerate; phosphoglycerate; ROS, reactive oxygen species; Ru5P, ribose-5-phosphate; RubP, ribulose-5-phosphate.

Lessons may be learned from studies on the mechanisms regulating the acquisition of N by C in non-symbiotic plants (review by Chaput et al., 2020). Sugars from photosynthesis or storage organs are known to be regulators of plant metabolism and plant gene expression (Rolland et al., 2006; Eveland and Jackson, 2012). Sucrose, transported from source tissues to sink organs, is first hydrolyzed either by Susy (to UDP-glucose and fructose) or by invertase (to glucose and fructose) before entering cellular metabolism, to be ultimately oxidized to CO_2_ through respiration. Multiple levels of sugar sensing have been characterized: a sucrose sensing pathway *via* a yet unknown sensor (Vaughn et al., 2002); the hexokinase pathway, which, independently of its glucose phosphorylation activity, has glucose sensing activity (Jang et al., 1997; Granot et al., 2013); a hexokinase-independent pathway, probably linked to the regulator of G-protein signaling (RGS1) located on the plasma membrane (Grigston et al., 2008; Urano et al., 2012); a glycolysis-dependent pathway downstream of hexokinase (Xiao et al., 2000); a trehalose pathway (Lunn et al., 2014); and a pathway related to the supply of carbon substrates to mitochondrial respiration (Aubert et al., 1996). As regards N acquisition, several investigations carried out in *Arabidopsis* roots have shown that the expression of the NO_3_^−^ transporter genes *NRT2.1* and *NPF6.3* was directly related to the concentration of glucose-6-phosphate in the roots (Lejay et al., 2003; Lejay et al., 2008). The use of 6-aminonicotinamide, an inhibitor of glucose-6-phosphate dehydrogenase (G6PDH) and 6-phosphogluconate dehydrogenase (6PGDH), two enzymes of the oxidative part of the pentose phosphate pathway (OPPP), as well as the use of a knockdown mutant for plastid 6-phosphogluconolactonase (PGL3), respectively made it possible to highlight the role of OPPP in the regulation of the expression of NRT2.1 and NPF6.3 (Lejay et al., 2008), as well as nitrate reductase (NR) and nitrite reductase (NiR; Bussell et al., 2013). Together with several studies highlighting the role of OPPP and sugars in the regulation of transporters involved in N acquisition (Oji et al., 1985; Bowsher et al., 1989; Bowsher et al., 1992; Neuhaus and Emes, 2000), these investigations supported the existence of an OPPP-dependent sugar signaling pathway for the regulation of plant N acquisition by roots. In plants, OPPP is the main NADPH regeneration pathway that helps maintain cellular redox balance, especially under oxidative stress. An increased flux through the OPPP results in an increased NADPH/NADP ratio and a better resistance to oxidative stress (Ralser et al., 2007), whereas the mutation of G6PDH, which determines the level of NADPH by controlling the flux of G6P that enters the OPPP, leads to a lower resistance to stress (Juhnke et al., 1996). In *Arabidopsis*, the recent demonstration of the regulation of *AtNRT2.1* by the redox status (Bellegarde et al., 2019) suggests that OPPP, *via* the regeneration of NADPH, could be the intermediary in C signaling. The chromatin factor HIGH NITROGEN INSENSITIVE9 (HNI9), encoded by a genetically identified regulatory locus of AtNRT2.1, was found to reduce the ROS levels under high, but not low, N provision (Widiez et al., 2011; Bellegarde et al., 2019). Interestingly, in *Arabidopsis*, the bZIP transcription factor ELONGATED HYPOCOTYL5 (HY5) was shown to be a shoot-to-root mobile systemic signal that mediates light promotion of root growth and NO_3_^−^ uptake *via* the activation of NRT2.1 (Chen et al., 2016). In the shoot, HY5 promotes indirectly carbon assimilation and translocation, whereas in the root, HY5-dependent upregulation of NRT2.1 and NO_3_^−^ uptake are favored by an increase in photosynthesis-derived sugars. Together with HNI9, HY5 is required for activation of the detoxification ROS program under high N (Bellegarde et al., 2019). The ability of HY5 to bind the promoter G-box of ROS-responsive genes and regulate de-etiolation in response to light and ROS suggests that HY5 could be involved in the crosstalk between sugars and redox state for the regulation of *NRT2.1* and several other NO_3_^−^ transporter genes by C through the OPPP (Chen et al., 2016; Gangappa and Botto, 2016; Chaput et al., 2020). This knowledge acquired in *Arabidopsis* on the regulation of NO_3_^−^ acquisition provides a basis to propose a hypothetical model for the regulation of symbiosis by photosynthesis (**Figure 8**). Interestingly, in soybean, the HY5 ortholog light-induced TGACG-motif binding factor 3/4 (GmSTF3/4) and FLOWERING LOCUS T (GmFTs) were shown to interdependently induce nodule organogenesis (Wang et al., 2021), supporting the idea that these transcription factors could also be part of the systemic regulation of symbiosis by C (**Figure 8**). OPPP also has a major role in symbiosis (**Figure 8**). NADPH is the primary redox cofactor that regulates the regeneration of glutathione and reduced ascorbate, which, in turn, act as secondary redox cofactors in the turnover, or even the detoxification, of reactive oxygen species (ROS) and reactive nitrogen species (RNS) (Noctor and Foyer, 1998; Apel and Hirt, 2004). Interestingly, ROS and nitric oxide (NO), as well as glutathione and homoglutathione, have been shown to be major regulators of symbiosis establishment and functioning (Pauly et al., 2006; Puppo et al., 2013; Berger et al., 2019). In mature nodules, NADPH oxidases (RBOHs) are major sources of H_2_O_2_ production, *via* superoxide anion dismutation (Marino et al., 2011; Arthikala et al., 2014). NR and electron transfer chains from both plant and bacterial partners significantly contribute to NO production in N_2_-fixing nodules (Sánchez et al., 2010; Horchani et al., 2011; Berger et al., 2021). The NO concentration is itself finely regulated by the phytoglobin Pgb1.1, whose function is to allow NO to exercise its signaling and metabolic intermediary functions at the different stages of the symbiosis without reaching toxic levels for the metabolism (Fukudome et al., 2016; Fukudome et al., 2019; Berger et al., 2020). In this reaction, NO is first oxidized to NO_3_^−^ by oxyPgb, which is converted to metPgb. MetPgb is then reduced by a MetPgb reductase (MetPgb-R) at the expense of NAD(P)H reducing power (Igamberdiev et al., 2006). Both S-sulfenylated and S-nitrosylated proteins, resulting from post-translational modifications generated by H_2_O_2_ and NO, have been detected during early interaction and in functioning nodules, linking ROS/NO production to redox-based protein regulation (Puppo et al., 2013). Thus, considered together, many studies allow to make functional links between carbon metabolism, the regeneration of NADPH and the regulation of redox status by OPPP, and the regulation of SNF by ROS and NO in mature nodules (**Figure 8**). However, this model remains highly speculative and demonstration of such mechanisms remains to be done.

## Multiple pathways are involved in the systemic control of symbiosis

**Figure 9:**
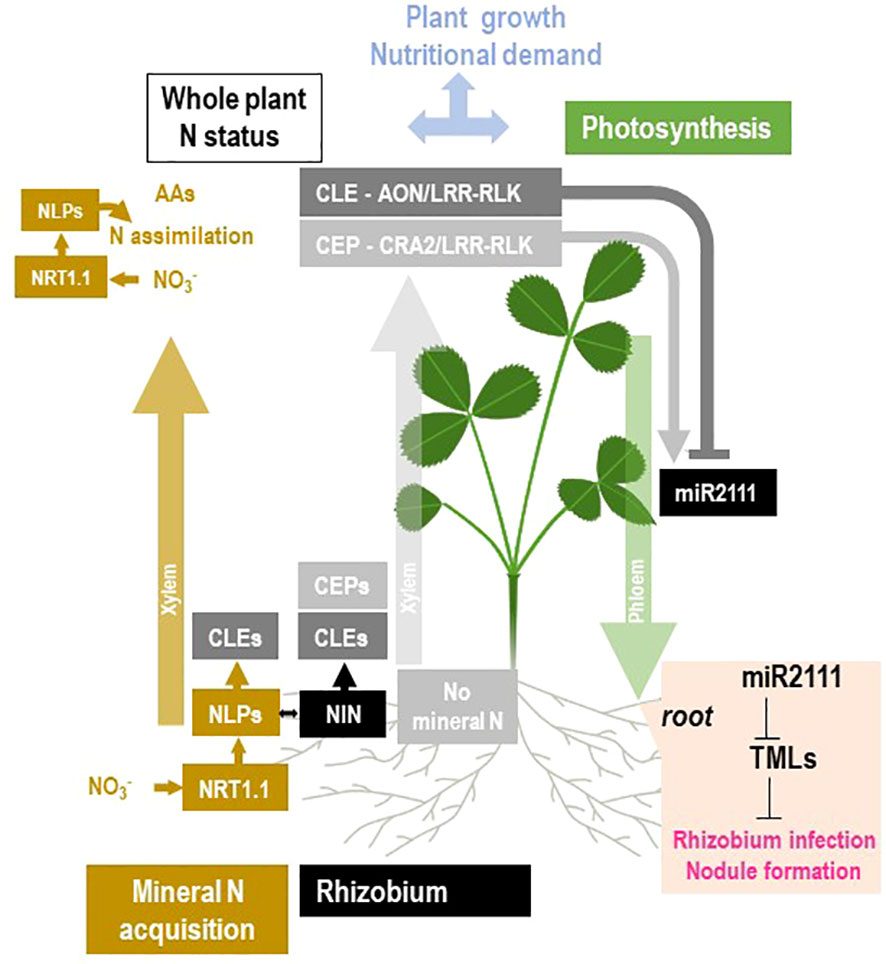
Schematic overview of the current knowledge of the molecular mechanisms potentially involved in the systemic regulation of nodule formation by the plant N status. The general framework of the figure is described in **Figure 2**. These mechanisms were initially related to the plant response to NO_3_^−^ (NRT1.1/NLPs module; in brown) or to the systemic control of nodule formation by the plant (CLE-AON/LRR-RLK and CEP-CRA2/LRR-RLK modules; respectively in dark and light gray). The mechanism related to the NO_3_^−^ response and to NO_3_^−^ assimilation is in brown. NO_3_^−^ fuels N assimilation and the production of amino acids. It also activates, through the action of the NO_3_^−^ transceptor NRT1.1 (NPF6.3/CHL1) and NLP transcription factors (MtNLP1 and LjNRSYM1), the accumulation of NO_3_^−^-responsive CLE peptides (MtCLE35, LjCLE-RS2, LjCLE-RS2, and Gm NIC1), as well as the genes encoding the enzyme of NO_3_^−^ assimilation (including NR and NiR). Rhizobium interaction triggers the accumulation of the NIN transcription factor, also related to NLP family, involved in the activation of CLE peptides in response to rhizobium (MtCLE12, MtCLE13). CLE peptides may be transported from the roots to the shoots through the xylem flow. In the shoots, the CLE peptide activation of AON/LRR-RLK receptors (MtSUNN, LjHAR1 forming possibly heterodimers with MtCLV2 and MtCRN or LjCLV2, and LjKLV) results in the downregulation of the level of miR2111 circulating in the phloem between shoot and roots. In the absence of mineral nitrogen, CEP peptides accumulate in the roots (MtCEP1 and MtCEP7) and are translocated to the shoot by the xylem. In the shoots, the CEP peptide activation of the CRA2/LRR-RLK receptor stimulates the miR2111 levels, antagonistically regulated by CLE-AON/LRR-RLK and CEP-CRA2/LRR-RLK pathways. In the roots, the miR2111 inhibits TML (by mRNA cleavage) that is actively repressing nodule formation. Consequently, CLE-AON/LRR-RLK and CEP-CRA2/LRR-RLK pathways respectively inhibits or promotes nodule formation.

In the last decade, significant discoveries allowed the characterization of receptors, peptides, and transduction pathways involved in the systemic control of nodule formation (**Figure 9**). Nevertheless, how these mechanisms are integrated at the whole plant level and contribute to the global phenotypes in response to variation of plant N demand and photosynthesis remains elusive. AON was shown to result in the inhibition of nodule formation by a pre-existing nodule (Kosslak et al., 1983; Kosslak and Bohlool, 1984; Mathews et al., 1989; Olsson et al., 1989; Kassaw et al., 2015). Evidenced by split-root experiments, this regulation involves systemic signaling between shoots and roots (Kosslak and Bohlool, 1984; Olsson et al., 1989; Kassaw et al., 2015). Pioneer genetic studies in several legume species allowed the identification of AON components (Caetano-Anollés and Gresshoff, 1991; Sagan et al., 1995; Wopereis et al., 2000; Chaulagain and Frugoli, 2021). AON mutants form generally more nodules than wild type and therefore display super/hyper nodulation phenotypes (Caetano-Anollés and Gresshoff, 1991; Sagan et al., 1995). Several recent reviews described in detail our current knowledge of the related molecular mechanisms (**Figure 9**; Chaulagain and Frugoli, 2021; Gautrat et al., 2021; Roy and Müller, 2022). AON involves CLV3-like 12-amino acid peptides (CLE) synthetized in roots, translocated by the xylem flux to the shoots, where they bind CLV3-like Leucine Rich Repeat Receptors Like Kinases LRR-RLK (**Figure 9**). *SUNN*, *HAR1*, and *NARK* loci encode these AON LRR-RLK in *M. truncatula*, *L.* japonicus, and soybean, respectively (Krusell et al., 2002; Nishimura et al., 2002; Searle et al., 2003; Schnabel et al., 2005). The receptors exist as homodimers or heterodimers formed with truncated co-receptors (LjCLV2, LjKLV in *L. japonicus* or MtCLV2 and MtCRN in *M. truncatula*; Miyazawa et al., 2010; Krusell et al., 2011; Crook et al., 2016). CLE peptides are encoded by large gene families in legumes and non-legume plants (Yamaguchi et al., 2016). The role of CLE peptides in the control of nodule number was demonstrated only for a few of them: MtCLE12, MtCLE13, and MtCLE35 in *M. truncatula* (Mortier et al., 2010; Mortier et al., 2012; Mens et al., 2020); LjCLE-RS1, LjCLE-RS2, and LjCLE-RS3 in *L. japonicus* (Okamoto et al., 2009; Nishida et al., 2016); and *Gm-RIC1* and *GmRIC2* in soybean (Reid et al., 2011a). The corresponding genes are upregulated in the roots in response to the rhizobium/nodule formation (**Figure 9**). The interaction of AON LRR-RLK receptor and CLE peptides activates the shoot-derived systemic inhibition of nodulation (**Figure 8**). A downstream component of AON LRR-RLK receptors is the small RNA miR2111 (**Figure 9**; Tsikou et al., 2018). miR2111 is synthetized as a precursor in the shoots, processed and translocated to the roots by the phloem. In the root, miR2111 post-transcriptionally represses TML1 and 2 genes, encoding ubiquitin ligases, resulting in the inhibition of nodule formation according to a still unknown mechanism (**Figure 9**; Magori et al., 2009; Takahara et al., 2013; Tsikou et al., 2018; Gautrat et al., 2019). Other signaling processes may also be implicated downstream of AON LRR-RLK receptors. In *L. japonicus*, the symbiosis establishment results in a HAR1-dependent upregulation of cytokinin synthesis in the shoots that is implicated in the regulation of nodule formation in roots (Sasaki et al., 2014). In addition, AON was associated in *M. truncatula* with a reduction of the shoot-to-root transport of auxin (van Noorden et al., 2006). Early studies revealed that AON mutants maintain the ability to form nodules under high NO_3_^−^ supply, demonstrating the role of AON in the control of nodulation by the plant N status (Carroll et al., 1985; Sagan et al., 1995). The resistance of nodulation to NO_3_^−^ in the *sunn* mutant of *M. truncatula* was related to a release of the N-satiety systemic repression (Jeudy et al., 2010). The response of *sunn* and wild-type roots to systemic N signaling during nodule formation was compared using split-root systems. A role of AON in the control of nodule formation by plant N demand was confirmed but AON-independent components were evidenced (Kassaw et al., 2015; Pervent et al., 2021). The role of AON in the regulation of nodulation by NO_3_^−^ was also discussed in *L. japonicus* (Nishida et al., 2020). Some CLE genes encoding peptides were found to be upregulated by NO_3_^−^ (MtCLE35, LjCLE-RS2, LjCLE-RS3, and Gm-NIC1) and to inhibit partial nodulation through the AON LRR-RLK receptor/miR2111/TML pathway (**Figure 9**; Okamoto et al., 2009; Nishida et al., 2016; Lebedeva et al., 2020; Mens et al., 2020; Moreau et al., 2021). In *M. truncatula*, only MtTML2 is downregulated in response to MtCLE35 overexpression, suggesting a specificity of the response to NO_3_^−^ as compared to the response to rhizobium/nodule formation (Moreau et al., 2021). For decades, AON was the unique identified systemic pathway controlling symbiotic development. Discovery of *M. truncatula TR185/cra2* mutants shaded the light on an additional pathway responsible for systemic activation of the root nodulation capacity (Bourion et al., 2014; Huault et al., 2014; Laffont et al., 2019). The mutants display highly branched root phenotype and modified responses to NO_3_^−^ in non-symbiotic conditions (Bourion et al., 2014; Huault et al., 2014). Their capacity to form nodules with rhizobium is dramatically impaired due to the absence of a systemic signaling originated from the shoot (Huault et al., 2014; Laffont et al., 2019). The *MtCRA2* gene, impaired in the mutants, encodes a Leucine-Rich Repeat Receptor-Like Kinase (LRR-RLK) present in shoots able to interact with peptides of the CEP family (C terminally encoded peptides; **Figure 9**). Both MtCEP1 and MtCEP7 peptides were found to activate the CRA2 systemic signaling, allowing nodulation (Laffont et al., 2019; Laffont et al., 2020). The two corresponding genes are upregulated in the root in response to the absence of mineral nitrogen and to rhizobium, suggesting a control of the pathway by both nitrogen status of the plant and infection by the bacteria. Intriguingly, MtCEP/MtCRA2 and the AON MtCLE/MtSUNN pathways share the downstream miR2111/TML component but act antagonistically on it (**Figure 9**). MtCEP/MtCRA2 stimulates the accumulation of miR2111 in the shoot to promote the cleavage MtTML transcript in the root, resulting in a stimulation of the root nodulation capacity (Gautrat et al., 2019; Gautrat et al., 2020). Nevertheless, the relative contribution of MtCEP/MtCRA2, MtCLE/MtSUNN, and possibly other unknown components in the control of nodulation by the N status of the plant is not well understood. Furthermore, the role, if any, of MtCEP/MtCRA2 on the response mature nodules to N demand and mineral N is not known because the mutant is impaired in nodule formation. Physiological and molecular characterization of *TR185/cra2* plants in non-symbiotic conditions described a N-limitation phenotype and the effect of the mutation on plant NO_3_^−^ acquisition and on root NO_3_^−^ transporters’ gene expression. AtCEPR1/AtCEPR2 orthologs of MtCRA2 as well as CEP peptides were identified in *Arabidopsis* (Tabata et al., 2014). A root target of systemic action of AtCEP1/CEPR1 is the high-affinity NO_3_^−^ transporter gene *AtNRT2.1*, known to be upregulated in response to the whole plant N deficiency. AtCEPD1 and AtCEPD2 (CEP downstream 1 and 2), two putative Class 3 glutaredoxins, might play the role of a systemic phloem signal from shoot to root to upregulate *AtNRT2.1* (Ohkubo et al., 2017). High-affinity NO_3_^−^ transporters are not the only targets of CEP/CEPR1 and CEP/CRA2 pathways both in *Arabidopsis* and in non-symbiotic *M. truncatula* plants, and root architecture was found to be strongly impaired in related mutants (Bourion et al., 2014; Delay et al., 2019; Chapman et al., 2020). How similar are the CEP receptors’ transduction pathways involved in the control of NO_3_^−^ uptake and root development in non-symbiotic conditions and the control of nodule formation in symbiotic conditions is not clearly understood. The role of CEPD proteins in the systemic regulation of nodulation downstream of CRA2 in *M. truncatula* was questioned (Gautrat et al., 2020). Although it cannot be excluded that CRA2 might be a component of the same mechanism adjusting either nodule formation, NO_3_^−^ acquisition, or root development to plant N demand, this remains to be demonstrated. Because the inactivation of CEP receptor genes has pleiotropic impacts on plant development and functioning, as well as mineral nitrogen acquisition, discriminating between direct and indirect impacts on the nodule formation phenotype in legumes is difficult. Nevertheless, the dramatic inhibition of nodulation observed in the *cra2* mutant cannot be simply explained as the result of the N-deficiency phenotype because plant N deficit stimulates rather than inhibits nodulation. To our knowledge, there is little convincing evidence for an active role of bacteria in the regulation of nodules by the holobiont N status (Lambert et al., 2020). However, several reports indicate that GlnD and PII, two bacterial regulatory components controlling nitrogen metabolism in bacteria, are required for symbiosis functioning, suggesting that the question might deserve more investigation (Arcondéguy et al., 1997; Yurgel et al., 2012; D’Apuzzo et al., 2015).

## Does the local sensing of NO_3_^−^ contribute to the control of symbiosis by plant N demand?

In addition to its role as a resource for downstream N-metabolite synthesis, NO_3_^−^ itself plays a role of signaling molecule in plant organs (review by Maghiaoui et al., 2020). In *Arabidopsis*, the use of null NR mutants’ NO_3_^−^-specific effects is independent of its reduction (Zhang et al., 1999; Wang et al., 2004). The complex mechanisms related to root NO_3_^−^ sensing begin to unravel in *Arabidopsis* (Maghiaoui et al., 2020). The NO_3_^−^ transporter AtNRT1.1 (NPF6.3/CHL1) plays the role of sensor and governs a wide range of response to NO_3_^−^ independently of its transport activity and NO_3_^−^ assimilation (Ho et al., 2009; Krouk et al., 2010; Bouguyon et al., 2015; Riveras et al., 2015; Maghiaoui et al., 2020). Other central players are some NLP transcription factors required for the induction of many target genes by NO_3_^−^ including those responsible for its reduction and assimilation (Castaings et al., 2009; Marchive et al., 2013; Guan et al., 2017; Liu et al., 2017; Liu et al., 2022). Several studies described the regulation in legumes of both nodule formation and functioning by NO_3_^−^ through the action of NLP proteins (**Figure 9**; Lin et al., 2018; Nishida et al., 2018; Moreau et al., 2021). In *M. truncatula*, MtNLP1, activated in response to NO_3_^−^, and MtNIN, required for nodulation in the presence of rhizobium, were shown to antagonistically interact for the transcriptional activation of key genes (**Figure 9**; Lin et al., 2018). NLP proteins (MtNLP1 and LjNRSYM1) were implicated in the transcriptional activation of NO_3_^−^-responsive genes, including *MtCLE35* and *LjCLE-RS2*, as well as *NR* and *NiR* genes (Nishida et al., 2018; Moreau et al., 2021). This local activation of *CLE* genes was shown to be associated with activating the LjHAR1/MtSUNN AON LRR-RLK-dependent systemic inhibition of nodulation in model legumes (**Figure 9**). Nevertheless, the biological significance of the hypothesis of systemic inhibition of nodulation activated locally by NO_3_^−^ remains elusive because (1) split-root studies rather suggest a regulation of nodulation by N demand related to downstream N-metabolite production at the whole plant level (Pervent et al., 2021) and (2) such mechanism does not explain inhibition of symbiosis by other N sources such as amino acids or NH_4_^+^ (Yamashita et al., 2019). Furthermore, because of their roles in the activation of NO_3_^−^ assimilation, an indirect impact of NLPs on downstream N-metabolite synthesis cannot be ruled out. The use of legume mutant background impaired in NO_3_^−^ reduction such as null NR mutants may unequivocally discriminate between a role of these NLPs in the inhibition of nodulation by NO_3_^−^ itself or by products of its assimilation. Finally, there are intriguing reports showing that, in some conditions, NO_3_^−^ may be required for optimal nitrogen fixation in mature nodules of *L. japonicus* through the action of specific nodule NO_3_^−^ transporters (Valkov et al., 2017; Valkov et al., 2020). This raised the hypothesis of a control of symbiotic activity by the NO_3_^−^ flux fueling the Pgb-NO respiration known to be active in the microoxic conditions of mature nodules (Horchani et al., 2011).

## Perspectives

The last decade yielded important knowledge on multiple mechanisms involved in the adjustment of the symbiotic capacity to the plant nutritional demand as a function of the plant environment. Because symbiosis allows the plant to acquire N from air at the expense of photosynthates, the plant N and C status are major drivers of these mechanisms. Local environmental conditions are tuning the adjustment of symbiosis activity to the whole plant nutritional status not only through the availability of N and C resources (mineral nitrogen, light, and CO_2_) but also by allowing or inhibiting the development and/or functioning of symbiotic organs. Plants continuously adapt to these conditions that are frequently heterogeneous in space and time. The nutritional demand is therefore necessarily integrated at the level of the whole plant, resulting in foraging responses either by stimulating symbiotic capacity (under N-deficit or eCO_2_ conditions) or by inhibiting it (under N-satiety or low-light conditions). These responses are activated by both systemic and local signaling pathways. The discovery of multiple pathways, acting simultaneously and targeting almost all aspects of nodule development and functioning, revealed not only the central role of the adjustment of the symbiotic capacity to the plant nutritional demand, but also its extraordinary complexity. However, the biological impact of these pathways and their relative role in the whole plant phenotype as a function of the environment is far to be understood.

Up to now, most investigations mainly focused on regulatory circuits controlling early plant–rhizobium interaction and nodule formation. Because the nodule formation process is associated with the activation of a large set of specific genes, earlier studies have predicted that specific symbiotic mechanisms may be operating in this control (Ruffel et al., 2008). However, the current knowledge prompted us to modulate this interpretation. Although the MtCEP/MtCRA2, the AON MtCLE/MtSUNN, or the NLP-related NO_3_^−^-responsive pathways target many specific symbiotic genes and development processes in legume plants, there is increasing evidence indicating that (1) legume mutants impaired in these pathways display non-symbiotic phenotypes often related to N nutrition, and (2) these pathways belong to families of pathways present in non-legumes regulating root development and mineral nitrogen acquisition in response to NO_3_^−^. The HY5 pathway was found to be important not only for the systemic regulation by light of NO_3_^−^ acquisition under non-symbiotic conditions but also for nodule formation under symbiotic conditions. A major challenge for future studies will be to revisit the plant phenotypes and discriminate between the pleiotropic consequences of mutations impairing the functions of these pathways. How much the impact of the Mtcra2 mutation on non-symbiotic functions (nitrogen limitation and root development) might influence the nodule formation phenotype of the mutant remains an open question. We do not know if, in NLP mutants (LjNRsym1 and Mtnlp1), the reduced activation by NO_3_^−^ of the NO_3_^−^ assimilation pathway, which is expected to lower the levels of downstream N metabolites, contributes to reduce the response of nodulation to NO_3_^−^. Discriminating between the effects of NO_3_^−^ itself and its assimilation on the regulation of nodulation will require appropriate strategies (NR mutants, for example). Although many progresses have been made in our understanding of AON, several studies suggest that some pieces of the puzzle in the control nodule formation by systemic signaling N demand remain unknown, particularly the mechanisms involved in the bacteroid differentiation and in the activation of nitrogen fixation in newly formed nodules (Pervent et al., 2021). Furthermore, although the last decade yields the discoveries of CLE and CEP peptides as well as miR2111, playing the role of signal molecules between root and shoot, the role of the other plant hormones in the systemic control of nodule formation by plant N demand remains to be clarified. Although ethylene has been implicated in the control of nodulation (particularly infection), its role in the plant response to N and C status through systemic signaling regulation deserves further investigation (Penmetsa et al., 2003; Prayitno et al., 2006; Zhu et al., 2020).

Less attention was made on the control of mature nodule development and functioning by the whole plant nutritional status. However, the N and C status of the plant may strongly determine mature nodule behavior, either by stimulating nodule expansion or by activating nodule senescence. Consistently with the tight integration of N and C signaling, the regulation of sucrose allocation to the nodule was associated with N-satiety and N-deficit systemic signaling, suggesting that the fueling symbiosis by C metabolites may contribute to a systemic N-demand signaling process. (Jeudy et al., 2010; Lambert et al., 2020). Supporting this hypothesis, transcripts encoding sucrose transporters likely responsible for the nodule acquisition of sucrose are targets of the N-demand systemic signaling pathway (Lambert et al., 2020). However, further studies are required to validate this model and its biological relevance. More globally, there is a lack of knowledge on mechanisms responsible for the coordination of symbiotic activity and photosynthesis. The last decade yielded the discovery of the role of the HY5 pathway in the control of nodule formation (Chen et al., 2016; Wang et al., 2021). However, questions related to biological significance and physiological impact of this pathway in the control of symbiosis deserve further investigations. The HY5 pathway was identified as a response to light, whereas most of the physiological data suggest a control of symbiosis by photosynthates allocated from the shoots to the roots. Furthermore, eCO_2_ stimulates symbiosis without any change in light and, therefore, independently of light-induced regulation of HY5. Up to now, most of the reported functions of HY5 relate to nodule formation and little is known about its role in mature nodule functioning. Whether additional function of HY5, or other mechanisms, is involved in the regulation of the symbiosis by photosynthesis and sucrose allocation by the plant remains to be further investigated. The discovery of the role of OPPP and the redox status in the regulation of plant NO_3_^−^ acquisition by photosynthesis suggests evaluating its role in the control of symbiosis in legumes. Although the critical role of the redox status (ROS and NOS) in symbiotic development and functioning has been clearly demonstrated, the possible role of the C metabolites’ allocation and the metabolites’ flux through the OPPP (providing reducing power necessary for the control of redox status) is an attracting hypothesis in the context of the regulation of symbiosis by the whole plant nutritional status. One of the major lessons of the last decade is our need to analyze integrated phenotypes of mutants impaired in regulatory components of symbiosis taking into account not only specific symbiotic function but also their possible interactions with whole holobiont development, and considering C and N economy as well as their impact on metabolic fluxes. Several efforts in this direction have been made: (1) modeling C and N exchanges as well as metabolic flux in the context of the symbiotic plant (Salon et al., 2009; Moreau et al., 2012; diCenzo et al., 2016; Schulte et al., 2021) and (2) investigating these regulations using split-root systems, which allows discrimination between local and inter-organ signaling (Ruffel et al., 2008; Jeudy et al., 2010; Laguerre et al., 2012; Lambert et al., 2020; Pervent et al., 2021).

A major biological role of these mechanisms relates to the whole symbiotic plant adaptation to local conditions impairing symbiotic activity (such as drought, salt, temperature, heavy metals, and flooding). As a sessile organism, the holobiont adapts to local stress and circumvents the plant N deficit resulting from local inhibition of SNF by stimulating nodule development in the root, allowing efficient symbiosis (Jeudy et al., 2010; Laguerre et al., 2012). This adaptation to satisfy the N demand is made by allocating preferentially C resources to more efficient roots at the expense of inefficient ones. In the context of climate change, soil conditions will be more heterogeneous and fluctuating than ever, resulting more frequently in local stresses (Dusenge et al., 2019). In addition, atmospheric ambient CO_2_ will increase, potentially modifying the conditions of plant C acquisition. A major challenge for plant science is to design strategies to select genotypes better adapted to these new conditions. The potential of legumes in this context has been highlighted because, unlike cereals acquiring mainly NO_3_^−^ as a N source, symbiotic N_2_-fixing legumes can benefit from elevated CO_2_ (Rogers et al., 2009). Despite the major threat of climate change in agriculture, changes in the equilibrium of C/N trade-offs in symbiotic crop legumes may be an opportunity for the selection of new genotypes able to better adapt to soil local constraints and display better competitivity as compared to other C3 non-symbiotic plants.

## Author contributions

All authors listed have made a substantial, direct, and intellectual contribution to the work and approved it for publication.
